# MitoQ Inhibits Human Breast Cancer Cell Migration, Invasion and Clonogenicity

**DOI:** 10.3390/cancers14061516

**Published:** 2022-03-16

**Authors:** Tania Capeloa, Joanna Krzystyniak, Donatienne d’Hose, Amanda Canas Rodriguez, Valery L. Payen, Luca X. Zampieri, Justine A. Van de Velde, Zohra Benyahia, Erica Pranzini, Thibaut Vazeille, Maude Fransolet, Caroline Bouzin, Davide Brusa, Carine Michiels, Bernard Gallez, Michael P. Murphy, Paolo E. Porporato, Pierre Sonveaux

**Affiliations:** 1 Pole of Pharmacology and Therapeutics, Institut de Recherche Expérimentale et Clinique (IREC), Université Catholique de Louvain (UCLouvain), 1200 Brussels, Belgium; tania.demiranda@uclouvain.be (T.C.); joannakrzystyniak@yahoo.co.uk (J.K.); amandacanasrodriguez@gmail.com (A.C.R.); valerypayen@hotmail.com (V.L.P.); luca.zampieri@uclouvain.be (L.X.Z.); justine.vandevelde@uclouvain.be (J.A.V.d.V.); zohra.benyahia@gmail.com (Z.B.); erica.pranzini@unifi.it (E.P.); thibaut.vazeille@uclouvain.be (T.V.); paolo.porporato@unito.it (P.E.P.); 2 Biomedical Magnetic Resonance Unit, Louvain Drug Research Institute (LDRI), Université Catholique de Louvain (UCLouvain), 1200 Brussels, Belgium; donatienne.dhose@uclouvain.be (D.d.); bernard.gallez@uclouvain.be (B.G.); 3 Department of Experimental and Clinical Biomedical Sciences Mario Serio, University of Florence, Via le Morgagni 50, 50134 Firenze, Italy; 4 Faculty of Sciences, Bology, Laboratoire de Biochimie et Biologie Cellulaire, University of Namur, Rue de Bruxelles 61, 5000 Namur, Belgium; maude.fransolet@unamur.be (M.F.); carine.michiels@unamur.be (C.M.); 5 IREC Imaging Platform (2IP), Université Catholique de Louvain (UCLouvain), 1200 Brussels, Belgium; caroline.bouzin@uclouvain.be; 6 IREC Flow Cytometry and Cell Sorting Platform, Université Catholique de Louvain (UCLouvain), 1200 Brussels, Belgium; davide.brusa@uclouvain.be; 7 MRC Mitochondrial Biology Unit, Department of Medicine, University of Cambridge, Hills Road, Cambridge CB2 0XY, UK; mpm@mrc-mbu.cam.ac.uk; 8 Department of Molecular Biotechnology and Health Science, Molecular Biotechnology Center, University of Turin, Via Nizza 52, 10126 Turin, Italy

**Keywords:** breast cancer, migration, invasion, clonogenicity, spheroids, metastasis, mitochondria, mitochondrial superoxide, MitoQ, mitochondria-targeted antioxidant

## Abstract

**Simple Summary:**

Solid tumors comprise metabolically hostile areas adjacent to metabolically friendly ones, and their distribution fluctuates in space and time. Selection pressure is maximal in hostile areas where cancer cells develop at least four survival strategies: to adapt themselves, to modify the microenvironment, to hibernate metabolically, and to escape. Escape marks the transition from a localized to an invasive tumor that may ultimately disseminate remotely, forming metastases. Based on the hypothesis that mitochondria are metabolic sensors that control these responses, we previously established that mitochondrial superoxide is a pro-metastatic intracellular signaling agent. Here, we tested MitoQ, a mitochondria-targeted ROS inactivator that already successfully passed Phase I safety clinical trials, as a potential inhibitor of the early steps of the metastatic cascade. Using human breast cancer cells as models in anticipation of preclinical and clinical assays, we report that MitoQ inhibits cancer cell migration, invasion, clonogenicity, sphere formation and spheroid stability.

**Abstract:**

To successfully generate distant metastases, metastatic progenitor cells must simultaneously possess mesenchymal characteristics, resist to anoïkis, migrate and invade directionally, resist to redox and shear stresses in the systemic circulation, and possess stem cell characteristics. These cells primarily originate from metabolically hostile areas of the primary tumor, where oxygen and nutrient deprivation, together with metabolic waste accumulation, exert a strong selection pressure promoting evasion. Here, we followed the hypothesis according to which metastasis as a whole implies the existence of metabolic sensors. Among others, mitochondria are singled out as a major source of superoxide that supports the metastatic phenotype. Molecularly, stressed cancer cells increase mitochondrial superoxide production, which activates the transforming growth factor-β pathway through src directly within mitochondria, ultimately activating focal adhesion kinase Pyk2. The existence of mitochondria-targeted antioxidants constitutes an opportunity to interfere with the metastatic process. Here, using aggressive triple-negative and HER2-positive human breast cancer cell lines as models, we report that MitoQ inhibits all the metastatic traits that we tested in vitro. Compared to other mitochondria-targeted antioxidants, MitoQ already successfully passed Phase I safety clinical trials, which provides an important incentive for future preclinical and clinical evaluations of this drug for the prevention of breast cancer metastasis.

## 1. Introduction

What distinguishes malignant tumors from benign tumors is their capability to become locally invasive and to ultimately metastasize. The metastatic switch is a discrete event marking the transition between a localized lesion and a systemic disease [1]. It results from the selection of metastatic progenitor cells in the primary tumor. To acquire metastatic capabilities, epithelial cancer cells first undergo an epithelial to mesenchymal transition (EMT), which notably provides resistance against detachment-induced cell death (anoïkis) and is associated with cell polarization, which facilitates motility [2,3]. Protrusions (encompassing filipodia and invadopodia), invaginations (comprising clathrin-coated pits) and adhesion modules supported by dynamic integrin–actin interactions are formed [4]. Following EMT, mesenchymal cells may further acquire the ability to migrate directionally towards blood and lymph vessels across the extracellular matrix (invasion), gain resistance to redox and shear stresses, immune evasion, and, ultimately, the capability to generate a new, secondary tumor from a single or from a small number of cells (stemness characteristics) [5,6]. Importantly, metastatic progenitor cells must possess all these characteristics together, explaining why they are a minority, including within the population of circulating tumor cells (CTCs) [7,8]. When a secondary lesion is established, pioneer cancer cells and their progeniture may revert, at least partially, to their initial phenotype. This process starts with a mesenchymal to epithelial transition (MET) [9].

From a metabolic standpoint, solid tumors are composed of metabolically hostile areas (characterized by hypoxia, limitations in nutrient bioavailability and waste accumulation) adjacent to metabolically friendly areas, and their repartition fluctuates over time and space. Adaptation and selection pressures are believed to be maximal in hostile microenvironments, where cancer cells have several survival options: switching metabolic activities, inducing angiogenesis, hibernating metabolically, and escaping. In all cases, this would imply the existence of metabolic sensors informing about the composition of the tumor microenvironment. Such systems include oxygen-dependent enzymes and redox-sensitive proteins that collaboratively activate transcription factors (such as hypoxia-inducible factors [HIFs] and nuclear factor-κB [NF-κB]), trigger the expression and secretion of cytokines (such as transforming growth factor-β [TGF-β]) and the expression of microRNAs (such as miR-200), and modify epigenetic marks [10,11,12]. Previous work by others and from our team further points to mitochondria as metabolic sensors [13,14]. For instance, Ishikawa et al. [15] previously showed that transferring mitochondria from metastatic progenitor cells to nonmetastatic cancer cells also transferred the metastatic phenotype. Using human cervix adenocarcinoma and mouse melanoma cells as models, we further observed that the Darwinian selection of highly metastatic cells sequentially involves enhanced tricarboxylic (TCA) cycle activities, increased mitochondrial superoxide (mtO_2_^−^) production, and activation of the TGF-β pathway at the level of src directly within mitochondria [16]. While, on the one hand, all experimental approaches that successfully increased mtO_2_^−^ production below cytotoxic levels also increased the metastatic activity of initially poorly metastatic cancer cells, on the other hand, inactivating mtO_2_^−^ with mitochondria-targeted antioxidant mitoTEMPO strongly repressed these activities [16]. For instance, mitoTEMPO completely prevented the occurrence of spontaneous metastases in mice bearing human triple-negative MDA-MB-231 breast tumors.

Several metabolic alterations can increase mtO_2_^−^ levels, and several pro-metastatic pathways in addition to the TGF-β pathway are sensitive to reactive oxygen species (ROS) [12], justifying our choice to directly inactivate mtO_2_^−^ for antimetastatic applications. In our opinion, mitochondrial selectivity is key, as general antioxidants unpredictably modulate (repress or induce) cancer metastasis [17,18].

**Figure 1:**
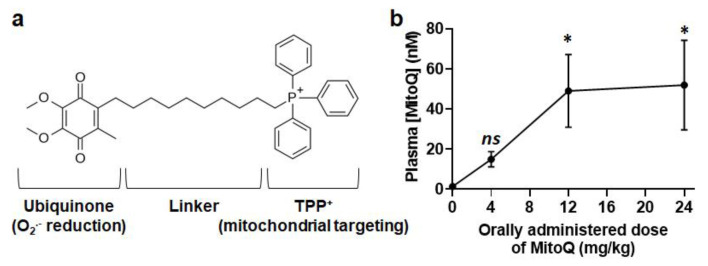
Determination of MitoQ levels in mouse plasma following per os administration. (**a**) Chemical formula of MitoQ showing the antioxidant coenzyme Q10 moiety, the linker and the positively charged triphenylphosphonium (TPP^+^) group that addresses the drug to mitochondria. (**b**) Female BALB/c mice received increasing doses of MitoQ per os, and blood was collected 4 h later for analysis using LC/MS/MS. The graph shows the plasma concentration of MitoQ in function of the administered dose (*n* = 9–14). All data are shown as means ± SEM. * *p* < 0.05; *ns*: *p* > 0.05 compared to control; by one-way ANOVA followed by Dunnett’s post hoc test (**b**).

To translate our finding in a potential clinical application, we decided to target mtO_2_^−^ with mitoquinol mesylate (MitoQ), a mitochondria-targeted antioxidant composed of coenzyme Q10 (the antioxidant moiety) covalently linked to triphenylphosphonium (TPP^+^, a lipophilic cation that ensures mitochondrial accumulation driven by the mitochondrial potential [Δψ]) by a 10-carbon alkyl chain (Figure 1a) [19]. A major motivation to test this drug in particular is that it already successfully passed Phase I safety clinical trials [20]. MitoQ rapidly crosses biological membranes and concentrates up to 100-fold at the matrix surface of the mitochondrial inner membrane [21]. There, compared to mitoTEMPO that acts as a superoxide-dismutase 2 (SOD2)-mimetic [22], MitoQ acts as a chain-breaking antioxidant that cycles between an ubiquinone form that captures some superoxide generated by the electron transport chain (ETC) and an ubiquinol form that acts as an antioxidant [20]. As it is believed to reduce superoxide down to water and to be recycled rather than destroyed in the end of the process, MitoQ may be particularly efficient at decreasing mtO_2_^−^ signaling. Even if the extent of uptake may be different due to differences in Δψ, it is expected to behave similarly within normoxic and hypoxic tumor areas because, contrary to endogenous coenzyme Q10, it is not oxidized by ETC Complex III [23]. These characteristics make of MitoQ a potential first-in-class drug to prevent cancer metastasis, which we tested here in vitro using highly metastatic human triple-negative breast cancer (TNBC) and HER2+ breast cancer cell lines. At clinically relevant doses, we report that MitoQ inhibits human breast cancer cell migration, invasion, clonogenicity, sphere formation and spheroid stability. It barely affects EMT, partially represses the expression of stemness-related genes, and exerts cytostatic effects at high yet clinically relevant doses. These encouraging results prompted us to perform preclinical assays in mice, which are disclosed in a companion paper in Cancers [24].

## 2. Materials and Methods

### 2.1. Chemicals

Mitoquinol mesylate (MitoQ) was produced as previously described [19]. Unless stated otherwise, all other chemicals were from Sigma-Aldrich (Overijse, Belgium). Equal volumes of solvent (DMSO) were used in control experiments.

### 2.2. Dosage of MitoQ in Mouse Plasma

MitoQ concentrations were measured in the plasma of 8-week-old female BALB/cAnNCrl mice (Charles River, Beerse, Belgium) 4 h after the administration of a single dose of MitoQ (from 0 to 24 mg/kg) by oral gavage, which corresponds to the half-life of MitoQ in rat kidneys after oral delivery [25]. Animals were anesthetized using a 0.1 mL/20 g intraperitoneal injection of ketamine: xylazine (87.5 mg/kg:12.5 mg/kg), and blood was collected via puncture of the facial vein. Plasma was isolated following centrifugation at 3500 rpm for 10 min. Organic extraction and MitoQ quantification using LC/MS/MS were performed as previously described [26]. Deuterated MitoQ (d_3_-MitoQ) was used as an internal standard.

### 2.3. Cells and Cell Culture

Triple-negative MDA-MB-231 human breast adenocarcinoma cancer cells were from Caliper (Mechelen, Belgium; catalogue #119369). HER2+ SkBr3 human breast adenocarcinoma cancer cells (catalogue #HTB-30), triple-negative MDA-MB-436 human breast adenocarcinoma cancer cells (catalogue #HTB-130) and MCF10A human nonmalignant breast epithelial cells (catalogue #CRL-10317) were from ATCC (Manassas, VA, USA). All cancer cell lines were originally derived from pleural effusions [27,28,29]. MDA-MB-231 and SkBr3 cells were routinely cultured in DMEM containing 4.5 g/L glucose and GlutaMax (Thermofisher, Erembodegem, Belgium; catalogue #10566016) with 10% FBS; MDA-MB-436 in IMDM containing GlutaMax (Thermofisher; catalogue #31980030) with 20% FBS; and MCF10A in DMEM:F-12 (Thermofisher; catalogue #11320033) with 5% horse serum, 1 mM CaCl_2_, 10 mM HEPES, 10 µg/mL insulin, 20 ng/mL epithelial growth factor (EGF) and 0.5 µg/mL hydrocortisone. Cells in culture were maintained at a subconfluent state in a humidified atmosphere with 95% room air and 5% CO_2_, 37 °C. Cell authenticities were routinely verified with a short tandem repeat (STR) test (Eurofins Genomics, Ebersberg, Germany).

### 2.4. Metabolic Assays

Oxygen consumption rates (OCRs) were determined on a Seahorse XF96 bioenergetic analyzer using the XF cell mito stress kit (Agilent Technologies, Machelen, Belgium), according to the manufacturer’s recommendations. The procedure is detailed in Appendix A. Glucose and lactate concentrations were measured in cell supernatants collected after 48 h of culture ± MitoQ, using specific enzymatic assays on a CMA600 analyzer (Aurora Borealis, Schoonebeek, The Netherlands), as previously described [30]. All data were normalized by total protein content (Protein Assay from Bio-Rad, Temse, Belgium).

### 2.5. Mitochondrial Potential

The mitochondrial potential (Δψ) was measured using the JC-10 Mitochondrial Membrane Potential Assay Kit (Abcam, Cambridge, UK; catalogue #ab112134), according to manufacturer’s recommendations. Briefly, MDA-MB-231 (10^4^ cells/well), SkBr3 (10^4^ cells/well), MDA-MB-436 (10^4^ cells/well) or MCF10A (10^5^ cells/well) cells were seeded in 96-well plates and treated for 48 h ± MitoQ. Cells were then washed twice and incubated with JC-10 (1× solution) for 45 min. Fluorescence intensities were measured at 490/525 nm and 540/525 nm of absorbance using a SpectraMax i3 spectrophotometer equipped with a MiniMax imaging cytometer (Molecular Devices, Munich, Germany).

### 2.6. Mitochondrial Superoxide

Mitochondrial superoxide levels were determined using electron paramagnetic resonance (EPR) with MitoTEMPO-H as a specific mitochondrial superoxide probe ± PEG-SOD2 to specifically assign signals to mitochondrial superoxide [31]. The procedure is detailed in Appendix A.

### 2.7. Cell Cycle

Cells were seeded in 6-well plates at a density of 200,000 cells/well and allowed to adhere overnight. They were then starved in 0.1% FBS medium for 24 h, and treated for 48 h ± increasing doses of MitoQ. Afterwards, cells were detached with trypsin-EDTA (0.05%) and centrifuged at 1200 rpm for 5 min. Culture medium was discarded, and the pellets were washed twice with cold PBS. Cells were fixed by adding 700 μL of ice-cold pure ethanol to 300 μL of cell suspension in PBS. They were then washed twice with 1 mL of Tris buffer with 0.2% (*v*/*v*) Triton X-100, and finally resuspended in 300 μL of PBS with RNase (0.2 mg/mL) and propidium iodide (5 μg/mL). At least 10^4^ events were acquired for each sample on a FACSCalibur flow cytometer (BD Biosciences, Erembodegem, Belgium), and the FlowJo software v10.8 (BD Biosciences) was used to analyze data according to DNA content.

### 2.8. Apoptosis and Necrosis Assays

Cells were seeded at 10^5^ cells per well in a 24-multiwell plate and allowed to adhere overnight. They were then treated ± increasing doses of MitoQ and cultured for 48 h. Apoptosis and necrosis were determined using the Annexin V Apoptosis Detection kit FITC (ThermoFisher; catalogue #88-8005-74) according to manufacturer’s recommendations. Profiles were determined by FACS on a Canto II flow cytometer (BD Biosciences). A minimum of 5000 events were acquired for each sample.

### 2.9. Immunocytochemistry

Cells cultured on glass coverslips were fixed in 4% formaldehyde, permeabilized with 0.1% Triton X-100 in PBS containing 0.1% Tween 20, and blocked with 5% BSA. Immunofluorescence staining was performed overnight using primary antibodies (Table S1). Secondary antibodies were an Alexa Fluor 488-conjugated goat anti-rabbit (ThermoFisher; catalogue #A-11034). Nuclei were stained with 4′,6-diamidino-2-phenylindole dihydrochloride (DAPI, 1 μg/mL, Sigma-Aldrich). Images were captured by structured illumination fluorescence microscopy using an ApoTome-equipped AxioImager.z1 microscope (Zeiss). Fluorescence intensity analysis was performed using the ImageJ software (NIH, Bethesda, MD, USA).

### 2.10. Cell Numbers

To probe the effects of MitoQ alone on cell numbers, 2500–10,000 cells per well were plated on 96-well plates and treated with increasing concentrations of MitoQ. At each time point, the number of cells per well was measured using a SpectraMax i3 spectrophotometer equipped with a MiniMax imaging cytometer.

### 2.11. Electron Microscopy

Electron microscopy images of MDA-MB-231 and SkBr3 cells treated ± MitoQ for 48 h were acquired on a TECNAI G² 20 LaB6 transmission microscope (Field Electron and Ion Company, Hillsboro, OR, USA) using a previously disclosed protocol [32].

### 2.12. Real-Time Quantitative PCR

Total mRNA from cells treated ± MitoQ for 48 h was extracted using the NucleoSpin RNA Kit (Filter Service, Eupen, Belgium). Total mRNA from primary tumors was extracted with Tri reagent (Brunschwig Chemie, Basel, Switzerland; catalogue #TR118). mRNAs were quantified by the Qubit BR dsRNA assay kit (Thermofisher), and mRNA integrity was evaluated on an Agilent 2100 Bioanalyzer with the RNA 6000 nano kit (Agilent). It was reverse-transcribed in complementary DNA using the High-Capacity cDNA Reverse Transcription Kit (ThermoFisher; catalogue #4368814) according to manufacturer’s protocol. Complementary DNA (500 ng) was amplified by real-time quantitative PCR (RT-qPCR) using the low ROX SYBR Master Mix dTTP Blue (Eurogentec, Seraing, Belgium) and primers listed in Table S2 on a ViiA 7 Real-Time PCR System (Thermofisher). All data were normalized to *β-actin* gene expression.

### 2.13. Western Blotting

For western blotting (WB), whole-protein extracts from cells were prepared using RIPA buffer (50 mM Tris pH 7.4, 150 mM NaCl, 1% Triton-X-100, 0.05% sodium deoxycholate, 1 mM EDTA, 0.1% SDS, protease inhibitor cocktail and PhosSTOP Phosphatase Inhibitor Cocktail [Sigma-Aldrich]), centrifuged at 10,000 g for 10 min, and quantified using the Bio-Rad Protein Assay. Proteins (50 µg for MDA-MB-231 and 100 µg for SkBr3 and MDA-MB-436 cells) were loaded into each lane of a 10–12% polyacrylamide gel in the presence of SDS, and were allowed to separate at 120 V for 90 min. PageRuler Prestained Protein Ladder (Thermofisher; catalogue #26616) was used as a molecular weight marker. The transfer of proteins onto nitrocellulose membranes was performed using an iBlot 2 Dry Blotting System (Thermofisher) with the 7 min P0 program. Membranes were then blocked using 5% (*w*/*v*) powdered milk for 1 h, and incubated overnight at 4 °C with a primary antibody (Table S1). β-actin served as a loading control. Immunodetection was performed at room temperature for 1 h using a horseradish peroxidase-conjugated goat anti-rabbit (The Jackson Laboratory, Sacramento, CA, USA; catalogue #111-035-003) and goat anti-mouse (The Jackson Laboratory; catalogue #115-035-003) as secondary antibodies in PBS-Tween containing 5% (*w*/*v*) milk or BSA. Detection was performed with ECL reagent (Amersham, Diegem, Belgium; catalogue #RPN2209), and protein bands were visualized and captured using ECL imager 600 (Amersham). They were analyzed using the ImageJ software. The original western blots see Figure S4.

### 2.14. Cell Migration

A scratch test was performed as previously shown [33] on cells treated ± MitoQ for 24 h. For each condition and each time point, pictures were taken in the same field, and the distance between wound edges was analyzed using the ImageJ software. Percentages of scratch closure were calculated with respect to the wounded area at 0 h.

### 2.15. Cell Invasion

Chemotaxis was assayed in a 48-well micro-chemotaxis chamber (Neuro Probe, Gaithersburg, MD, USA; catalogue #AP48) equipped with a 8 µm diameter porous polycarbonate membrane (Neuro Probe; catalogue #PFB8) coated with 5 µg/mL of fibronectin, according to manufacturer’s instructions. Briefly, MDA-MB-231 (20,000 cells/well), SkBr3 (40,000 cells/well) or MDA-MB-436 (80,000 cells/well) cells were seeded in the upper compartment in culture media deprived of FBS; and 0.2% (*v*/*v*) FBS-containing medium (for MDA-MB-231 and MDA-MB-436 cells) or 10% (*v*/*v*) FBS-containing medium supplemented with 10 ng/mL of human epithelial growth factor (EGF; PreproTech, London, UK; catalogue #AF-100-15; for SkBr3 cells) were used as chemoattractants. Cells were allowed to invade through the membrane overnight. Membranes were then washed with PBS, fixed in methanol, and stained with crystal violet (0.23% *v*/*v*). Pictures were acquired on an Axiovert 40 CFL microscope equipped with an MRC camera. Quantification was performed using the Image J software.

### 2.16. Clonogenic Assays

For adherent assays, MDA-MB-231 and SkBr3 cells were pretreated for 48 h ± MitoQ, and seeded (1000 to 2000 cells/well) in 6-well plates. After colony formation (2 weeks), cells were fixed and stained with 0.5% crystal violet in a 10% ethanol solution, for 30 min. Colonies were washed with water and counted. Results are expressed as surviving fraction (SF), where SF = #colonies/plating efficiency (PE).

For soft agar colony formation assays, 0.4% Seaplaque soft agar (Lonza, Verviers, Belgium) was diluted with DMEM containing 10% FBS and was covered by a second 0.3% soft agar layer in which 500 MDA-MB-231, SkBr3 or MDA-MB-436 cells were embedded. Complete DMEM culture medium ± MitoQ was added with 2× the final concentration. After 20 days, colonies were counted using an Axiovert 40 CFL microscope (Zeiss) equipped with an MRC camera.

### 2.17. Spheroids

To test the effects of MitoQ on sphere formation, 10^4^ MDA-MB-231, SkBr3 or MDA-MB-436 cancer cells were grown in suspension using 10 cm dishes coated with Polyhema in stem cell medium DMEM/F-12 (Thermofisher; catalogue #11320033) supplemented with 100 units/mL penicillin, 100 µg/mL streptomycin, 20 µL/mL B27 (Thermofisher; cat #17504044), 20 ng/mL human EGF (PeproTech; catalogue #AF-100-15), 10 ng/mL basic fibroblast growth factor (bFGF; PeproTech; catalogue #100-18B) and 2.5 mg/mL insulin. When spheres reached around 100 µm in diameter, they were washed with PBS and dissociated with accutase (Stem Cell Technologies, Saint Égrève, France; catalogue #07920) in order to have single cells again. At the third passage, dissociated spheres were treated ± MitoQ for 4 days. Images were captured on an Axiovert 40 CFL microscope equipped with an MRC camera. To determine the expression of stemness-associated genes, dissociated spheres were treated ± MitoQ 250 nM for 48 h, collected, washed with PBS and processed for RT-qPCR.

To test the effects of MitoQ on sphere stability, mature spheroids [34] were prepared by seeding 5000 MDA-MB-231, SkBr3 or MDA-MB-436 cells from dissociated spheres/well in ultra-low attachment 96-well plates (Corning, Tewksbury, MA, USA; catalogue #7007) in stem cell medium supplemented as described above. After overnight formation, mature spheroids were treated ± MitoQ for 4 to 8 days. Spheroid growth and size were monitored using an Axio Observer.z1 live-cell phase contrast microscope (Zeiss). Images were analyzed using Image J. Spheroid cores were delineated and quantified (pixels area) with the ‘Analyze’ and ‘Measure’ functions of the software. Data are expressed as percentages relative to spheroid core areas before treatment.

### 2.18. Statistics

All results are expressed as means ± standard error of the mean (SEM) for *n* independent observations. Error bars are sometimes smaller than symbols. Outliers were identified using Dixon’s Q test. Data were analyzed using GraphPad Prism 8.4.3 (San Diego, CA, USA). Student’s *t* test, one-way ANOVA with Dunnett’s post hoc test, and two-way ANOVA with Dunnett’s or Tukey’s post hoc tests were used where appropriate. *p* < 0.05 was considered to be statistically significant.

## 3. Results

### 3.1. Determination of a Biologically Relevant Dose Range of MitoQ

For the design of phenotypic assays and in anticipation of future preclinical assays, we first determined a biologically relevant dose range of MitoQ. Four hours after the oral delivery of a single dose (which corresponds to the half-life of MitoQ in rodents [25,35]), free MitoQ reached ~50 nM in mouse plasma, with a plateau starting from an administered dose of 12 mg/kg (Figure 1b). For comparison, in humans, oral dosing at 1 mg/kg resulted in a plasma concentration of ~50 nM of MitoQ ~ 1 h after delivery [20]. Considering that its use for the prevention of metastasis would imply a chronic treatment and taking into account that MitoQ, as a lipophilic cation, accumulates in tissues up to levels of 100 to 700 nmol/kg (wet weight) following oral delivery to mice [36], we decided to investigate doses of MitoQ ranging from 100 to 500 nM.

### 3.2. MitoQ Represses Oxidative Phosphorylation and Mitochondrial Superoxide Production by Metastatic Human Breast Cancer Cells

To test whether MitoQ can prevent the early steps of metastasis in breast cancer cells in vitro, we selected human triple-negative MDA-MB-231 breast adenocarcinoma cancer cells, human HER2+ SkBr3 breast adenocarcinoma cancer cells and human triple-negative MDA-MB-436 breast adenocarcinoma cancer cells as the main models. All three cell lines were originally derived from pleural effusion [27,28,29], attesting their metastatic capabilities. Spontaneously immortalized human MCF10A normal epithelial breast cells [37] served as controls. We started our characterization with metabolic assays.

**Figure 2:**
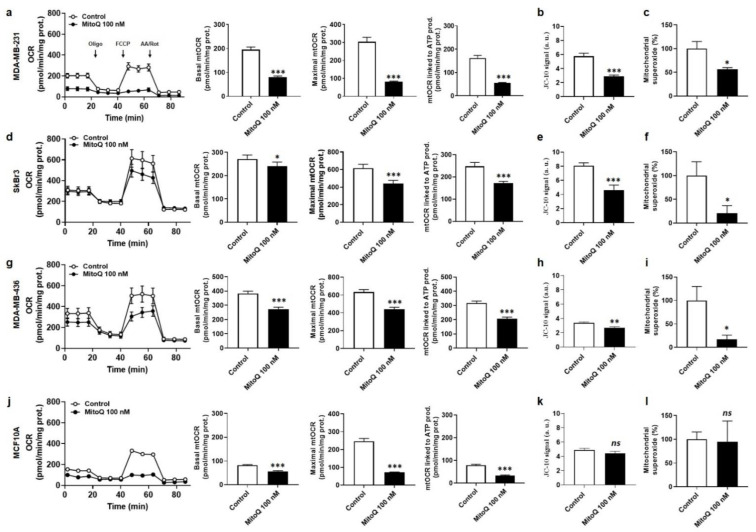
MitoQ selectively represses mitochondrial superoxide production by human breast cancer cells. Cells were treated ± MitoQ 100 nM for 48 h. (**a**) The oxygen consumption rate (OCR) of MDA-MB-231 cells was measured using Seahorse oximetry. The graph represents OCR measurements over time with the sequential addition of oligomycin, FCCP, and rotenone (Rot) together with antimycin A (AA). From Seahorse traces, basal, maximal and ATP-linked mitochondrial oxygen consumption rates (mtOCRs) were calculated (*n* = 8–18). (**b**) The mitochondrial potential (Δψ) of MDA-MB-231 cells was measured using JC-10 (*n* = 16). (**c**) Mitochondrial superoxide (mtO_2_^−^) levels were measured using electron paramagnetic resonance (EPR) with MitoTEMPO-H as a selective mtO_2_^−^ sensor ± PEG-SOD2 (*n* = 6). (**d**) Seahorse oximetry as in a, but using human SkBr3 breast cancer cells (*n* = 29–41). (**e**) Δψ measurement as in b, but using SkBr3 cells (*n* = 16). (**f**) Determination of mtO_2_^−^ levels as in c, but using SkBr3 cells (*n* = 4). (**g**) Seahorse oximetry as in a, but using human MDA-MB-436 breast cancer cells (*n* = 16). Note that SEMs are smaller than symbols in the left graph showing oximetry traces. (**h**) Δψ measurement as in b, but using MDA-MB-436 cells (*n* = 8). (**i**) Determination of mtO_2_^−^ levels as in c, but using MDA-MB-436 cells. (*n* = 4). (**j**) Seahorse oximetry as in a, but using nonmalignant MCF10A human breast epithelial cells (*n* = 18–24). (**k**) Δψ measurement as in b, but using MCF10A cells (*n* = 8). (**l**) Determination of mtO_2_^−^ levels as in c, but using MCF10A cells (*n* = 3). All data are shown as means ± SEM. * *p* < 0.05, ** *p* < 0.01, *** *p* < 0.001 compared to control; *ns*: *p* > 0.05 compared to control; by Student *t*-test (**a**–**l**).

In MDA-MB-231 cancer cells, treatment with MitoQ at 100 nM for 48 h decreased basal, maximal and ATP-linked mitochondrial oxygen consumption rates (mtOCRs) measured using Seahorse oximetry (Figure 2a), as well as the mitochondrial potential (Δψ) measured with JC-10 (Figure 2b). This was associated with a reduction in mtO_2_^−^ levels measured using electron paramagnetic resonance (EPR) with MitoTEMPO-H as a selective mtO_2_^−^ sensor and pegylated SOD2/MnSOD [31] for control (Figure 2c). Representative EPR experiments are displayed in Figure S1. Similar effects were observed using SkBr3 cancer cells (Figure 2d–f) and MDA-MB-436 cancer cells (Figure 2g–i), which both experienced decreased mtOCR, a decreased Δψ and lower mtO_2_^−^ levels in response to 100 nM of MitoQ for 48 h. Comparatively, in the same treatment conditions, MitoQ decreased basal, maximal and ATP production-linked mtOCRs, but not Δψ nor mtO_2_^−^ levels, in MCF10A nonmalignant breast cells (Figure 2j–l), unraveling the selective effects of MitoQ on cancer cells.

### 3.3. MitoQ Is Cytostatic for Human Breast Cancer Cells

**Figure 3:**
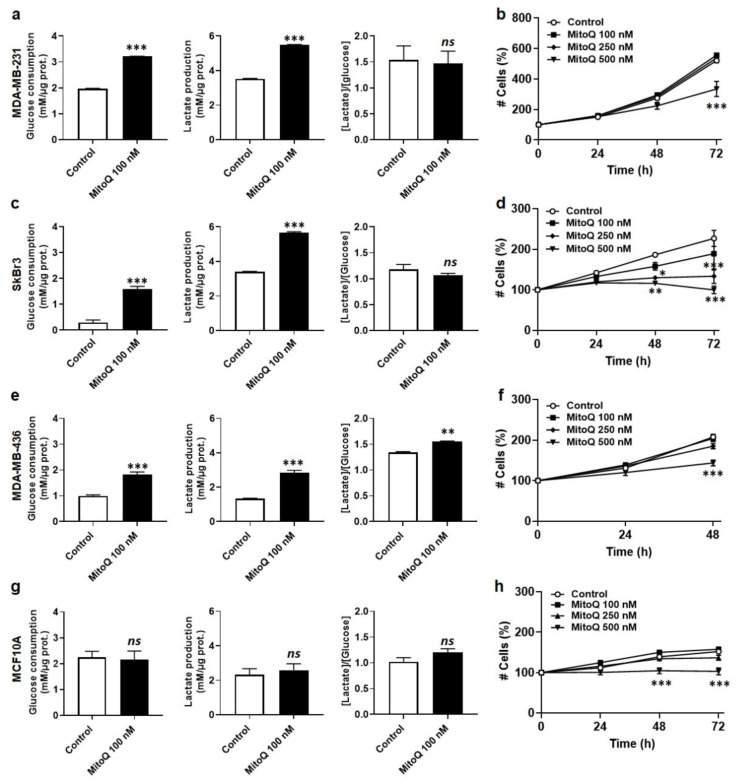
MitoQ increases glucose consumption and lactate release by human breast cancer cells, and is cytostatic at doses ≥ 250 nM. (**a**) MDA-MB-231 cells were treated ± MitoQ 100 nM for 48 h. Glucose consumption (left), lactate production (middle) and the lactate/glucose ratio (right) were then determined using enzymatic assays on a CMA600 analyzer (*n* = 3 all). (**b**) Viable MDA-MB-231 cells were counted on a SpectraMax i3 spectrophotometer at the indicated time points after treatment with increasing doses of MitoQ (*n* = 4). (**c**) Enzymatic measurements of glucose and lactate consumption as in a, but using SkBr3 cells (*n* = 10). (**d**) SkBr3 cell viability was determined as in b (*n* = 8). (**e**) Enzymatic measurements of glucose and lactate consumption as in a, but using MDA-MB-436 cells (*n* = 10). (**f**) MDA-MB-436 cell viability was determined as in b (*n* = 8). (**g**) Enzymatic measurements of glucose and lactate consumption as in a, but using MCF10A normal epithelial breast cells (*n* = 4). (**h**) MCF10A cell viability was determined as in b (*n* = 8). All data are shown as means ± SEM. * *p* < 0.05, ** *p* < 0.01, *** *p* < 0.001 compared to control; *ns*: *p* > 0.05 compared to control; by Student *t*-test (**a**,**c**,**e**,**g**) or 2-way ANOVA with Tukey’s post hoc test (**b**,**d**,**f**,**h**).

For cancer cells known to be highly adaptive metabolically, a normal survival response to oxidative phosphorylation (OXPHOS) inhibition is an enhanced glycolytic flux. Accordingly, MDA-MB-231 cancer cells responded to the treatment with 100 nM MitoQ for 48 h by increasing glucose consumption and lactate release, measured using a CMA600 enzymatic analyzer (Figure 3a). Their glycolytic efficiency (lactate/glucose ratio) was unchanged. Despite activation of this rescue pathway, dose- and time-dependent assays revealed that MitoQ reduced MDA-MB-231 cell number starting at 500 nM for a 72 h treatment (Figure 3b). Similar effects were observed in SkBr3 (Figure 3c,d) and MDA-MB-436 (Figure 3e,f) cells, except that cell numbers started to decrease 48 h after the administration of a single dose of 250 nM of MitoQ for SkBr3 cells and 48 h after the administration of a single dose of 500 nM of MitoQ for MDA-MB-436 cells. For nonmalignant MCF10A human breast epithelial cells, no significant changes were observed for glucose consumption and lactate production levels (Figure 3g). Cell numbers were stable up to 250 nM of MitoQ for 72 h, but decreased 48 h after a single dose treatment with 500 nM of MitoQ (Figure 3h). Note that the effects of MitoQ were cytostatic rather than cytotoxic, as cell numbers never decreased below plated numbers (Figure 2b,d,f,h), and no significant differences in cell apoptosis or necrosis were observed for any of the tested MitoQ concentrations upon annexin-V/PI staining (Figure S2a). Rather, higher doses of MitoQ generally blocked the cell cycle in the G0/G1 phase (Figure S2b).

Dose-dependent decreases in cell numbers matched with dose-dependent decreases in Δψ (Figure S3a), while glycolytic compensation reached its maximum in all cell lines (Figure S3b). Electron microscopy revealed intravesicular mitochondria accumulating in MDA-MB-231 and SkBr3 cells treated with 500 nM MitoQ (Figure S3c), which suggested mitochondrial damage and the activation of mitophagy.

### 3.4. MitoQ Partially Represses the Expression of Mesenchymal Marks by Human Breast Cancer Cells

Having established that low, nanomolar doses of MitoQ decreased mtO_2_^−^ levels in breast cancer cells, we next sought the determine the phenotypic consequences of MitoQ treatment on the early steps of the metastatic cascade. Because EMT precedes and favors metastasis [38], we started by exploring mesenchymal marks in the three model cancer cell lines.

**Figure 4:**
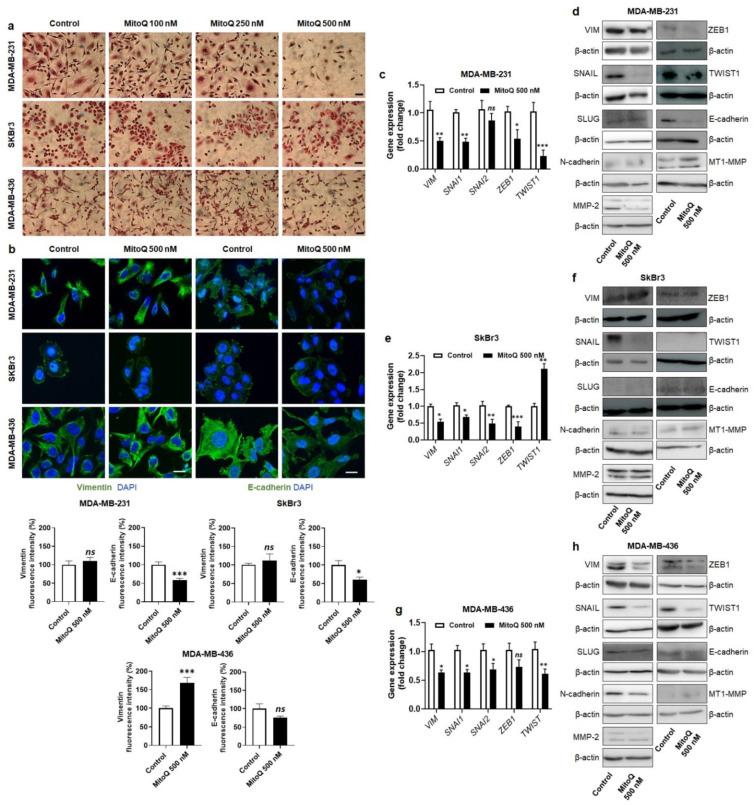
MitoQ has mitigated effects on the epithelial to mesenchymal transition (EMT) of human breast cancer cells. Cells were treated ± MitoQ for 48 h. (**a**) Representative immunocytological pictures where MDA-MB-231, SkBr3 and MDA-MB-436 cells are stained with hematoxylin and eosin. Bars = 1 mm. (**b**) Cells were stained with primary antibodies anti-vimentin and anti-E-cadherin (green fluorescence), and nuclei were stained with DAPI (blue). Representative pictures are shown on top, and the graphs on the bottom show the fluorescence intensity for MDA-MB-231 (*n* = 9–12), SkBr3 (*n* = 9–12) and MDA-MB-436 (*n* = 11-12) cells. Bars = 20 µm. (**c**) mRNA expression of EMT markers vimentin (*VIM*), SNAIL (*SNAI1*), SLUG (*SNAI2*), *ZEB1* and *TWIST1* in MDA-MB-231 cancer cells treated ± 500 nM MitoQ for 48 h (*n* = 6–9). (**d**) Western blots (WBs) of the corresponding proteins with β-actin as a loading control. (**e**) mRNA expression in c, but in SkBr3 cells (*n* = 3–9). (**f**) WBs as in d, but using SkBr3 cells. (**g**) mRNA expression in c, but in MDA-MB-436 cells (*n* = 6–9). (**h**) WBs as in d, but using MDA-MB-436 cells. All data are shown as means ± SEM. * *p* < 0.05, ** *p* < 0.01, *** *p* < 0.001 compared to control; *ns*: *p* > 0.05 compared to control; by Student *t* test (**b**,**c**,**e**,**g**).

Although MDA-MB-231, SkBr3 and MDA-MB-436 are of epithelial origin, these adenocarcinoma cell lines all have a mesenchymal phenotype (Figure 4a), as expected for metastatic breast cancer cells isolated from pleural effusions in patients [27,28,29]. MitoQ (500 nM, 48 h) had a rather discrete effect, if any, on the mesenchymal state of the three cell lines (Figure 4a). Immunofluorescent staining showed that MitoQ had no effect (MD-MB-231 and SkBr3 cells) or significantly increased (MDA-MB-436 cells) the expression of mesenchymal marker vimentin, while the expression of epithelial marker E-cadherin was either unchanged (MDA-MB-436 cells) or significantly decreased (MD-MB-231 and SkBr3 cells) (Figure 4b). More evident clues were provided when analyzing the expression of EMT markers/effectors. In MDA-MB-231 cells, MitoQ indeed decreased the mRNA expression of vimentin (*VIM*), SNAIL1 (*SNAI1*), *ZEB1* and *TWIST1*, whereas SLUG (*SNAI2*) transcription was not significantly altered (Figure 4c). At the same time point, SNAIL, ZEB1, TWIST1, and matrix metalloproteinase-2 (MMP-2) protein expression was decreased (Figure 4d and Figure S4a). In line with immunocytology data, the protein expression of E-cadherin was decreased too. These changes were not observed at doses of MitoQ lower than 500 nM. In SkBr3 cells, MitoQ (500 nM, 48 h) repressed the mRNA expression of all analyzed EMT markers except *TWIST1*, whose transcription was increased (Figure 4e). At the same time point, only SNAIL protein expression was decreased (Figure 4f and Figure S4b). SLUG and TWIST1 proteins were not expressed, and E-cadherin was barely detectable. In MDA-MB-436 cells, MitoQ (500 nM, 48 h) reduced the mRNA expression of EMT markers *VIM*, *SNAI1*, *SNAI2* and *TWIST1* (Figure 4g), as well as the protein expression of VIM, SNAIL, TWIST1, N-cadherin and MMP-2 (Figure 4h and Figure S4c). We concluded that MitoQ barely affects the mesenchymal state of human breast cancer cells having previously undergone EMT in patients. Only SNAIL protein expression was consistently decreased in the three cell lines.

### 3.5. MitoQ Inhibits Human Breast Cancer Cell Migration and Invasion

**Figure 5:**
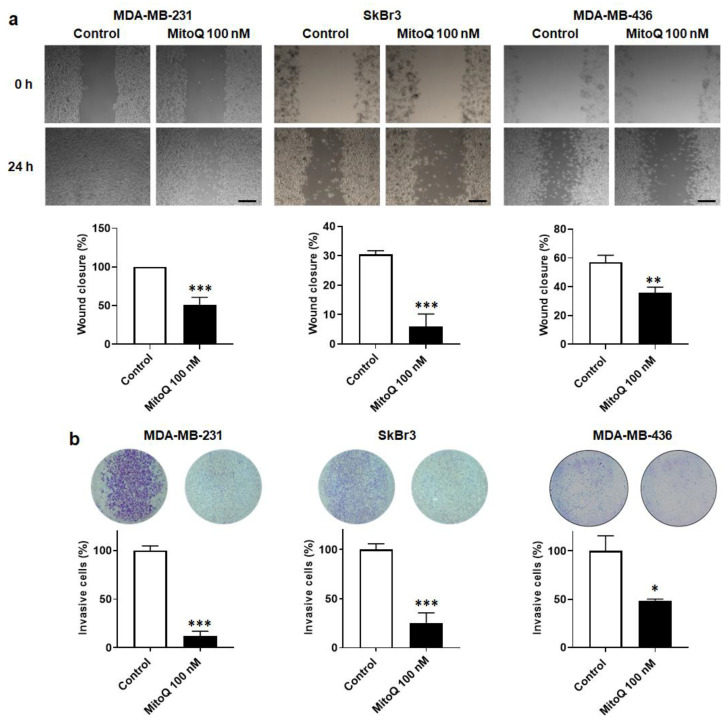
MitoQ represses human breast cancer cell migration and invasion. Cells were treated for 48 h ± MitoQ (100 nM). (**a**) MDA-MB-231 (left, *n* = 6), SkBr3 (middle, *n* = 3–8) and MDA-MB-436 (right, *n* = 10–13) cancer cell migration over 24 h was determined using a scratch assay. Representative pictures are shown on top and quantification graphs on the bottom. Bars = 50 µm. (**b**) MDA-MB-231 (left, *n* = 3), SkBr3 (middle, *n* = 3) and MDA-MB-436 (right, *n* = 3) cancer cell invasion was quantified in a Boyden chamber assay. Representative images are shown together with overnight invasion data. All data are shown as means ± SEM. * *p* < 0.05, ** *p* < 0.01, *** *p* < 0.001 compared to control; by Student *t*-test (**a**,**b**).

Along with EMT, metastatic cancer cells must acquire migratory and invasive capabilities to metastasize [5]. MDA-MB-231, SkBr3 and MDA-MB-436 cells possess these capabilities, which were strongly reduced upon treatment with MitoQ (100 nM, 48 h). Cell migration was assayed in scratch tests, where MitoQ reduced wound closure by ~40 to ~80% (Figure 5a). In transwells, MitoQ inhibited invasion by ~50 to ~90% (Figure 5b), depending on the cell line.

### 3.6. MitoQ Represses Human Breast Cancer Cell Clonogenicity, Sphere Formation and Spheroid Stability

**Figure 6:**
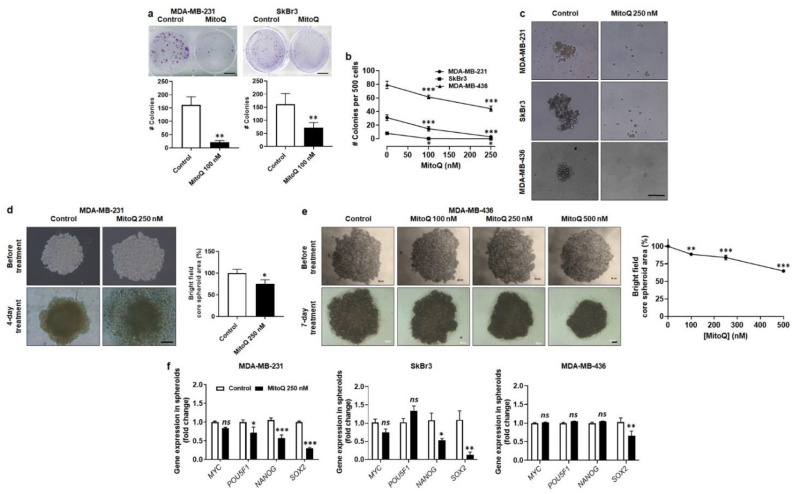
MitoQ represses human breast cancer cell clonogenicity, sphere formation and spheroid stability. (**a**,**b**) Cells were pretreated for 48 h with the indicated doses of MitoQ. (**a**) Clonogenic assay using adherent MDA-MB-231 (left, *n* = 6) and SkBr3 (right, *n* = 9) cells. Bars = 1 cm. (**b**) Clonogenic assay on soft agar using MDA-MB-231 (*n* = 16), SkBr3 (*n* = 16) and MDA-MB-436 (*n* = 8) cells. (**c**) Shown are representative images of MDA-MB-231, SkBr3 and MDA-MB-436 spheroid formation over 4 days in the presence or not of 250 nM MitoQ. Bar = 250 µm. (**d**) Representative images of mature MDA-MB-231 spheroids before and after 4 days of treatment ± 250 nM MitoQ are shown on the left (Bar = 250 µm). On the right, the graph represents spheroid size determined using the bright field mode of phase contrast microscope (*n* = 9–11). (**e**) As in d, but using MDA-MB-436 cells and a treatment of 7 days (*n* = 6 all; bars = 50 µm for images on the top and 200 µm for images on the bottom). (**f**) mRNA expression of cancer stem cell markers *MYC*, *POU5F1* (Oct4), *NANOG* and *SOX2* in MDA-MB-231 (left, *n* = 3–7), SkBr3 (middle, *n* = 4–6) and MDA-MB-436 (right, *n*= 4–8) spheres treated for 48 h ± 250 nM MitoQ. All data are shown as means ± SEM. * *p* < 0.05, ** *p* < 0.01, *** *p* < 0.001 compared to control; *ns*: *p* > 0.05 compared to control; by Student *t* test (**a**,**d**,**f**) or one-way ANOVA followed by Dunnett’s post hoc test (**b**,**e**).

To establish a secondary tumor in a distal organ, cancer cells must further possess stem cell characteristics [5]. In adherent conditions, MDA-MB-231 and SkBr3 cells were equally clonogenic, which was largely inhibited upon treatment with MitoQ (100 nM, 48 h) (Figure 6a). MDA-MB-436 were not clonogenic on plastic. In suspensions on soft agar, MDA-MB-436 were more clonogenic than MDA-MB-231 and SkBr3 cells, rendering them less sensitive to MitoQ (Figure 6b). Indeed, while 250 nM of MitoQ completely abrogated the clonogenicity of both MDA-MB-231 and SkBr3, the clonogenicity of MDA-MB-436 cells was only reduced by ~45% in the same conditions.

After 4 days of treatment, 250 nM of MitoQ also inhibited the formation of spheres from either MDA-MB-231, SkBr3 or MDA-MB-436 cells, which was assayed by delivering MitoQ during sphere formation (Figure 6c). SkBr3 spheres were not stable and did not yield mature [34] spheroids. Conversely, TNBC cell lines produced mature spheroids, which were significantly destabilized by MitoQ (Figure 6d,e). This effect was dose-dependent, with MDA-MB-231 spheroids (Figure 6d) being more sensitive to MitoQ than MDA-MB-436 spheroids (Figure 6e). Of note, unlike cancer cells, MCF10A are immortalized nonmalignant human breast epithelial cells that are not clonogenic and do not form spheroids.

The global effects of MitoQ on spheroids could involve different processes than mere inhibition of cancer cell proliferation. Indeed, in line with clonogenic assays (Figure 6a,b), the expression of stemness-related genes was decreased in MitoQ-treated spheres (Figure 6f). As for EMT-related genes, the response was partial, with decreased *POU5F1* (Oct4), *NANOG* and *SOX2* expression in MDA-MB-231, decreased *NANOG* and *SOX2* expression in SkBr3 and decreased *SOX2* expression in MDA-MB-436 cells.

Collectively, we concluded that MitoQ has the capacity to inhibit all major metastatic traits that we tested in vitro. Our next aim was to test this drug in preclinical mouse models of metastatic breast cancer, which is disclosed in a companion paper [24].

## 4. Discussion

To our knowledge, no specific anticancer treatment exists that prevents metastatic dissemination, making it a clinical priority [39]. Here, in anticipation of in vivo preclinical [24] and clinical assays, we report that MitoQ, a mitochondria-targeted antioxidant that already passed Phase I safety clinical trials [20], decreases mtO_2_^−^ levels in three different human cancer cell lines. It has mitigated effects on EMT, but clearly inhibits breast cancer cell migration, invasion, clonogenicity, sphere formation and sphere stability in vitro. These phenotypes that were repressed represent early steps of metastasis [5,40].

Malignant tumors are distinguished from benign ones by their capacity to invade and metastasize, which can take time to occur in vivo. Consequently, some cancer types are often already metastatic at diagnosis (e.g., melanoma), generally owing to a discrete, poorly symptomatic nature of the primary tumor; and others, such as prostate cancer, slowly evolve to the metastatic state. The main reason why we choose human breast cancer cell lines as a model in this translational study is that, although most breast cancer patients are diagnosed at the premetastatic stage, a majority of them relapse with local cancer recurrence and/or distant metastasis despite therapy [41]. Incidence rates are the highest in TNBC and HER2+ subtypes. In our opinion, frequent early diagnosis, a high predictability to metastasize, (some) latency and a very poor patient 5-year survival rate after the onset of metastasis (grade IV) in this type of cancer offer appropriate conditions to test drugs interfering with metastatic dissemination.

Using human cervix adenocarcinoma and mouse melanoma cells, a cause–consequence relationship has previously been established linking mtO_2_^−^ production to the metastatic progenitor cell phenotype [16]. On the one hand, in vitro or in vivo selection to foster invasion and metastasis was associated with increased mtO_2_^−^ production, which stayed beyond cytotoxic levels. Mechanistically, metastatic progenitor cells were characterized by an unbridled activity of the TCA cycle with the transfer of an excessive amount of electrons to the ETC, creating an overload manifested by increased electron leak and superoxide production [16]. On the other hand, experimentally triggering ETC electron leak and superoxide production by bottlenecking either of the ETC complexes (I to IV) using partial inhibition also increased the metastatic phenotype in vitro and in vivo [16]. Hence, transferring to naïve cancer cells mitochondria presenting a mtDNA defect responsible for mtO_2_^−^ production also transferred the metastatic phenotype [15]. The present study extends the scope of these findings to human breast cancer cells isolated from pleural effusion, which also presented high mtO_2_^−^ levels associated with migratory, invasive, clonogenic and sphere formation capabilities. Comparatively, nonmalignant human breast epithelial cells had barely detectable mtO_2_^−^ levels, as determined using EPR with MitoTEMPO-H as a selective sensor [31], pegylated SOD2 as a control ensuring specific superoxide quantification [31,42] and MitoQ as a selective antioxidant [43].

Several different mitochondrial defects and behaviors can promote metastasis [44]. Similarly, one can expect that several downstream pro-metastatic pathways are activated by mtO_2_^−^. One of them is the TGF-β pathway that can be activated at the level of src directly within mitochondria [16]. For activation, mitochondrial H_2_O_2_ acts as an intermediate [16] that can oxidize src on a specific cysteine residue (Cys277), thereby triggering src homodimerization and autophosphorylation on Tyr416 [45]. A long list of other pro-metastatic pathways, including the HIF and NF-κB pathways, are ROS-inducible [46], but information is lacking regarding their activation by mtO_2_^−^ Here, our objective was not to define these pathways (this will be the topic of a follow-up study), but, rather, to build foundations for translating these findings in clinical applications. Considering that the multiplicity of potential targets upstream and downstream of mtO_2_^−^ would increase the probability of compensatory mechanisms, we decided to directly target mtO_2_^−^.

Several mitochondria-targeted antioxidants exist, among which MitoQ differs by its mechanism of action. Indeed, contrary to drugs such as MitoVitE and MitoTEMPO that act as SOD-mimetics and are irreversibly oxidized by mtROS [47,48], MitoQ acts as a chain-breaking antioxidant that cycles between an oxidized ubiquinone form that captures mtO_2_^−^ and a reduced ubiquinol form that acts as an antioxidant [20]. It is therefore capable of completely reducing superoxide to water. As previously suggested by others [49], our observation that MitoQ also decreased the OCR and the Δψ of human breast cancer cells further suggests a competition between MitoQ and coenzyme Q10, whereby MitoQ would actively extract electrons from the ETC but would not be oxidized by Complex III [23]. MitoQ accumulation in mitochondria is known to increase with the Δψ [50], which we believe provides selectivity for metastatic progenitor cells compared to other cancer cells and host cells, as long as they have a high ETC activity.

Within a range of biologically relevant concentrations (100 to 500 nM) [20,37] (see also reference [24]), MitoQ strongly decreased human breast cancer cell migration, invasion, clonogenicity, sphere formation and spheroid stability. Its effects on EMT and on the mRNA expression of stem cell markers were more mitigated. Indeed, among stemness-related genes, only *SOX2* expression was decreased in spheres from all three cell lines and, among EMT markers, only SNAIL expression was decreased at the protein level in all tested cell lines. This is interesting, as *SOX2* transcription [51] and SNAIL expression [52] have been reported to be SOD2-sensitive, and SNAIL overexpression alone is sufficient to generate tumor-initiating breast cancer cells [53]. For the other tested EMT factors in our study, there was a limited correlation between mRNA and protein data, suggesting the existence of post-translational influences. Interestingly, the relevance of EMT for breast cancer metastasis has previously been questioned by Bill and Christophori [54] who concluded that the transient nature of EMT and the existence of migration modes different from mesenchymal migration make it difficult to ascertain the clinical importance of EMT for breast cancer metastasis. Others proposed that cells having undergone partial instead of complete EMT have a higher degree of plasticity, are more likely to gain stemness properties and, therefore, have the highest potential to become metastatic progenitor cells [55,56]. If so, and this would fit with the preservation of E-cadherin expression and the barely altered cell morphology in our model cell lines, then partial inhibition of EMT by MitoQ could participate in the repression of the pro-metastatic phenotype as a whole. The exploration of this hypothesis in mouse models of breast cancer is the topic of a companion paper [24] in *Cancers*.

## 5. Conclusions

Conclusively, our in vitro investigation validates MitoQ as an effective drug to inhibit the early steps of the metastatic process in human breast cancer cells. At biologically relevant doses, MitoQ collectively decreased cell migration and invasion, as well as clonogenicity, sphere formation and spheroid stability, which depend on stemness. These encouraging results support additional preclinical and clinical efforts to develop MitoQ as a preventive treatment against breast cancer metastasis.

## Data Availability

All data are contained within the article and Supplementary Materials.

## Supplementary Materials

The following supporting information can be downloaded at: https://www.mdpi.com/article/10.3390/cancers14061516/s1, Figure S1: Representative electron paramagnetic resonance (EPR) experiments for the specific measurement of mitochondrial superoxide; Figure S2: MitoQ is cytostatic for human breast cancer cells and immortalized nonmalignant human breast epithelial cells; Figure S3: MitoQ dose-dependently depolarizes mitochondria in human breast cancer cells; Figure S4: Uncropped western blot images; Table S1: Antibodies used for western blotting and immunocytochemistry; Table S2: Primers used in quantitative PCR.

## Appendix A. Detailed Materials and Methods

### Appendix A.1. Seahorse Oximetry

MDA-MB-231 (10^4^ cells/well), SkBr3 (10^4^ cells/well), MDA-MB-436 (10^4^ cells/well) and MCF10A (10^5^ cells/well) were plated on XF96 culture plates 16 h before experiments in DMEM containing 10% FBS, and treated ± MitoQ for 48 h. On the day of analysis, culture media were replaced by DMEM containing 10 mM glucose, 2 mM glutamine, 1.85 g/L NaCl, 3 mg/L phenol red, pH 7.4. Cells were incubated for 1 h in a CO_2_-free incubator before analysis. Sequentially, basal OCR was acquired without treatment; ATP-linked OCR after the addition of 1 µM of ATP synthase inhibitor oligomycin; maximal OCR after mitochondrial potential disruption using 1 µM of ionophore carbonyl cyanide-4-(trifluoromethoxy)phenylhydrazone (FCCP); and non-mitochondrial OCR after the addition of 0.5 µM of Complex I inhibitor rotenone together with 0.5 µM of Complex III inhibitor antimycin A. All data were normalized by total protein content (Bio-Rad Protein Assay; catalogue #5000006) measured right after oximetry. Mitochondrial OCRs (mtOCRs) were calculated by subtracting non-mitochondrial OCRs to the corresponding basal, maximal and ATP-linked OCRs.

### Appendix A.2. Mitochondrial Superoxide

EPR measurements of superoxide levels were performed using a Bruker (Kontich, Belgium) EMX-Plus spectrometer, operating in X-band (9.85 GHz), equipped with a PremiumX ultra-low noise microwave bridge and a SHQ high-sensitivity resonator. Typical settings were: microwave power: 20 mW; attenuation: 10 dB; modulation frequency: 100 kHz; modulation amplitude: 0.1 mT; time constant: 20.48 ms; conversion time: 22.58 ms; sweep width: 0.15 mT. Briefly, cells were treated ± MitoQ for 48 h, harvested and resuspended in PBS at 2 × 10^6^ cells/mL. The experimental mixture was prepared with cell suspension, 1 mM DTPA and 150 µM MitoTEMPO-H. It was then aspirated into gas-permeable polytetrafluoroethylene (PTFE) tubing that was placed in a quartz tube opened at both ends. The tube was directly inserted inside the EPR cavity that was heated at 310 K with air during all experiments. EPR measurements were performed 3 min after incorporation of the probe within the cell mixture, and acquisitions were continued until 15 min. To specifically measure superoxide contribution to the EPR signal, each experiment was repeated replacing 2.5 µL PBS by 2.5 µL of PEG-SOD2 (stock: 4000 U/mL). Computer simulations were performed using the Bruker Xenon Spin fit program. All data were normalized by cell numbers (trypan blue assay). Net superoxide production was calculated by subtracting the double integration values of the MitoTEMPO-H spectrum with PEG-SOD2 to the control value at time point 15 min.
