# Supplementary material for: MitoQ Inhibits Human Breast Cancer Cell Migration, Invasion and Clonogenicity
Source: Cancers (Basel). 2022 Mar 16;14(6):1516. doi: 10.3390/cancers14061516 (PMC8946220; doi:10.3390/cancers14061516)
Supplement: Supplementary file 1 [file cancers-14-01516-s001.zip › cancers-1644610-supplementary.pdf]

Supplementary Material: MitoQ Inhibits Human Breast Cancer Cell Migration, Invasion and Clonogenicity

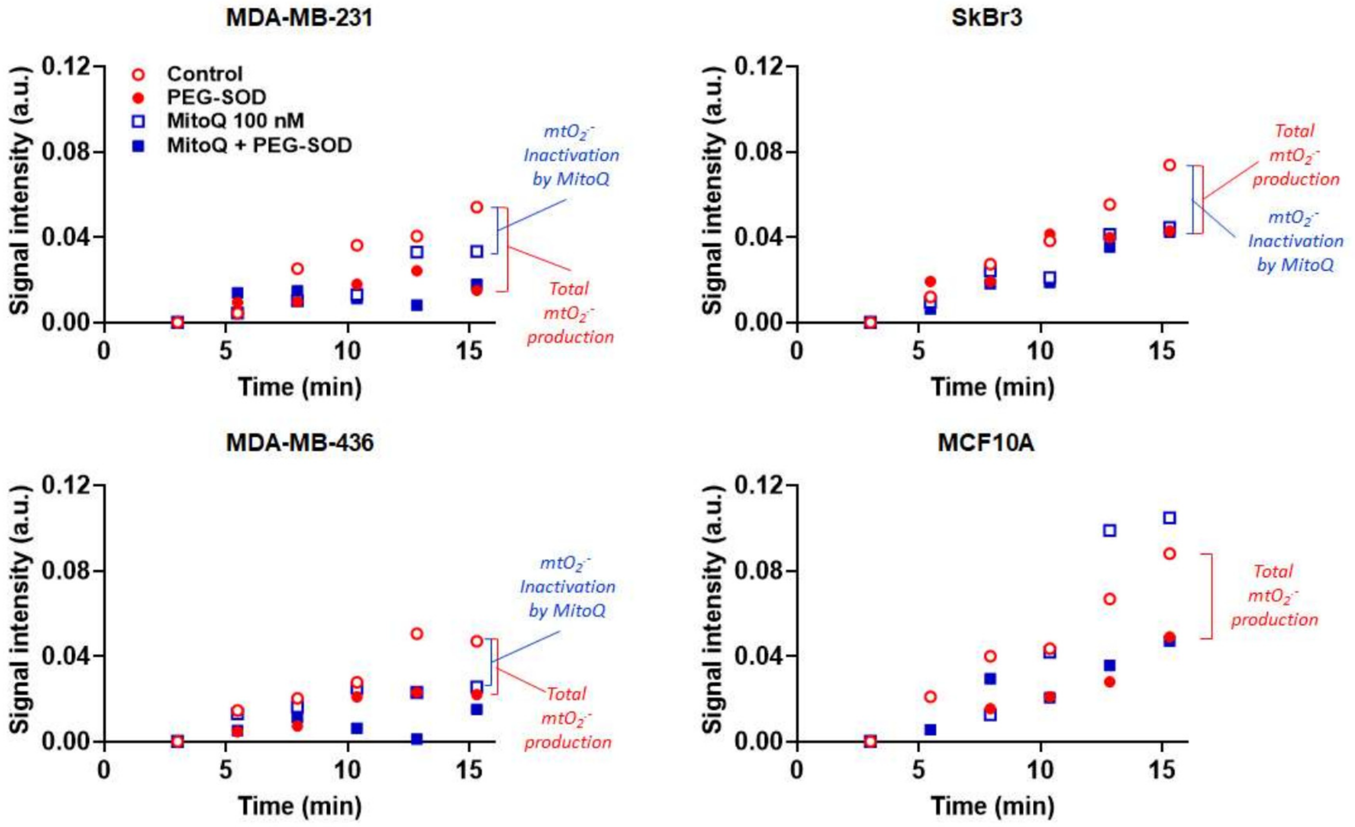

Figure S1. Representative electron paramagnetic resonance (EPR) experiments for the specific measurement of mitochondrial superoxide. Graphs report on mitochondria-targeted MitoTempo-H probe oxidation over time in human MDA-MB-231, SkBr3 and MDA-MB-436 breast cancer cells, as well as in nonmalignant human MCF10A epithelial cells, treated for 48 h ± 100 nM MitoQ, and then treated ± 10 U PEG-SOD2 at the time of the assay. Symbols represent the signal intensity of recorded EPR spectra during time for each treatment condition after double integration of the peaks. Relative superoxide levels (arbitrary units) are measured at time = 15 min. EPR signals in the control condition (open red symbols) represents total mtROS production. The difference between control (open red symbols) and the PEG-SOD2 condition (closed red symbols) represents total mitochondrial superoxide production, i.e., the contribution of mitochondrial superoxide to the oxidation of MitoTempo-H. The difference between control (open red symbols) and the MitoQ condition (open blue symbols) represents the amount of mtROS inactivated by MitoQ. The difference between the MitoQ condition (open blue symbols) and the MitoQ + PEG-SOD2 condition (closed blue symbols) represents the amount of mitochondrial superoxide that MitoQ did not inactivate.

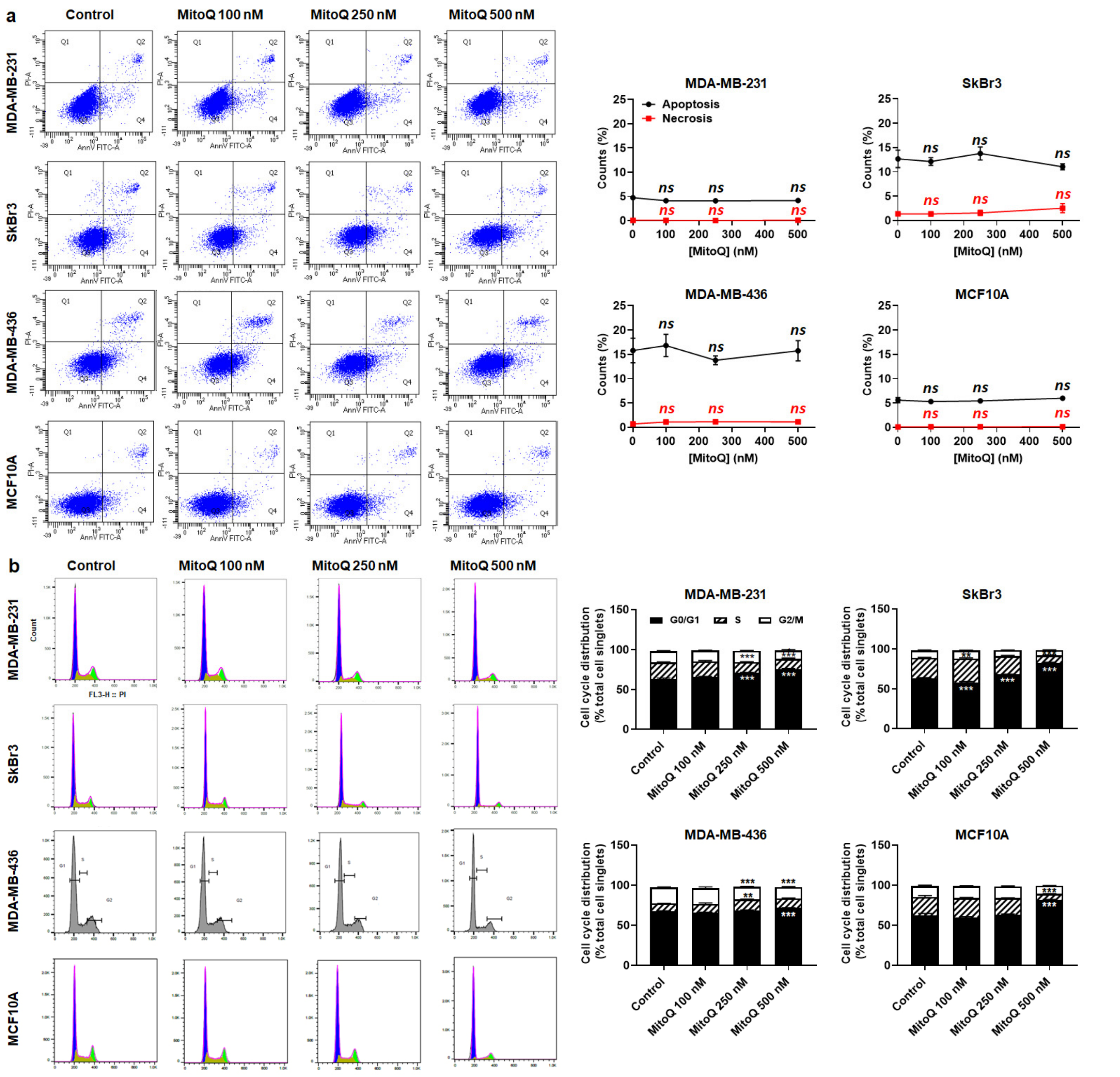

Figure S2. MitoQ is cytostatic for human breast cancer cells and immortalized nonmalignant human breast epithelial cells. (**a**–**b**) Cells were treated for 48 h by increasing doses of MitoQ. (**a**) Apoptosis and necrosis were evaluated using annexin V-FITC/PI staining followed by flow cytometry. Graphs on the left show the flow cytometric data of annexin V-FITC/PI double staining, where apoptotic cells are located in Q4 and necrotic cells in Q2. Graphs on the right show quantifications ($n = 4$–8). (**b**) Cell cycle progression was determined using propidium iodide staining followed by flow cytometry. Representative traces are shown on the left, and the graphs display the relative amount of cells in each phase of the cell cycle (total = 100%; $n = 4$ all). All data are shown as means ± SEM. * $p < 0.05$, ** $p < 0.01$, *** $p < 0.001$ compared to control; *ns*: $p > 0.05$ compared to control; by one-way with Dunnett's post hoc test (**a**) and 2-way ANOVA with Dunnett's post-hoc test (**b**).

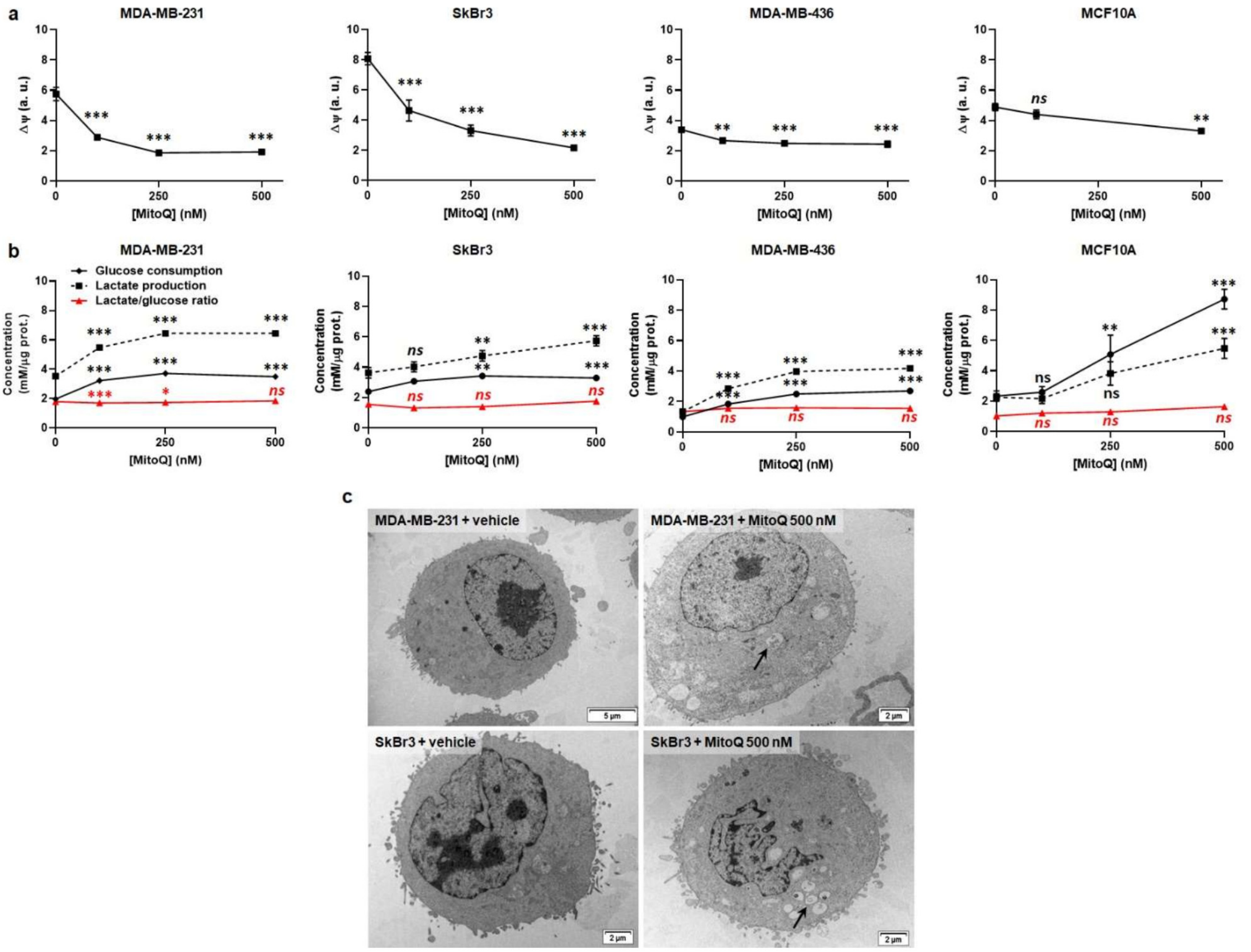

Figure S3. MitoQ dose-dependently depolarizes mitochondria in human breast cancer cells. Human breast cancer cells were treated for 48 h with the indicated doses of MitoQ. (**a**) The mitochondrial potential (Δψ) of MDA-MB-231 (left) (n = 16), SkBr3 (middle left) (n = 16), MDA MB-436 (middle right) (n =16) and MCF10A (right) (n = 5–8) cells was measured using JC-10 on a SpectraMax i3 spectrophotometer. (**b**) Glucose consumption, lactate production and lactate/glucose ratio were measured in MDA-MB-231 (left, n = 3), SkBr3 (middle left, n = 5–6), MDA-MB-436 (middle right, n = 4) and MCF10A (right, n = 4) cells using enzymatic assays on a CMA600 analyzer. (**c**) Shown are representative electron microscopy pictures of MDA-MB-231 and SkBr3 cancer cells treated ± 500 nM MitoQ for 48 h (bars = 2 μm or 5 μm as mentioned). Arrow shows typical vesicles containing mitochondria. All data are shown as means ± SEM. * $p < 0.05$, ** $p < 0.01$, *** $p < 0.001$ compared to vehicle; *ns*: $P > 0.05$ compared to vehicle; by one-way ANOVA with Dunnett's post-hoc test (**a**) or by 2-way ANOVA with Dunnett's post-hoc test (**b**).

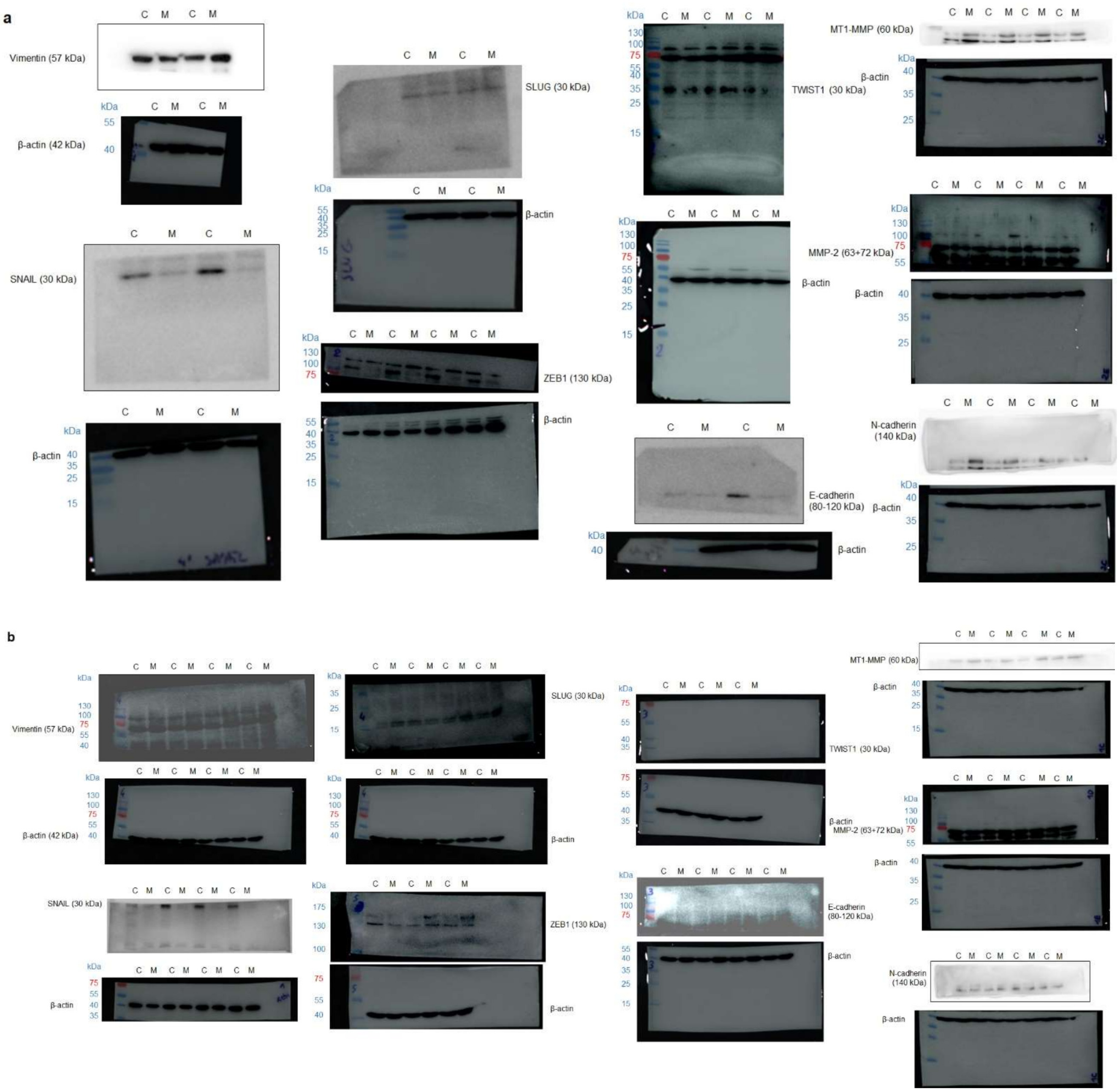
a
Vimentin (57 kDa)
β-actin (42 kDa)
SNAIL (30 kDa)
β-actin
SLUG (30 kDa)
β-actin
ZEB1 (130 kDa)
β-actin
TWIST1 (30 kDa)
β-actin
E-cadherin (80-120 kDa)
β-actin
MT1-MMP (60 kDa)
β-actin
MMP-2 (63+72 kDa)
β-actin
N-cadherin (140 kDa)
β-actin
b
Vimentin (57 kDa)
β-actin (42 kDa)
SNAIL (30 kDa)
β-actin
SLUG (30 kDa)
β-actin
ZEB1 (130 kDa)
β-actin
TWIST1 (30 kDa)
β-actin
E-cadherin (80-120 kDa)
β-actin
MT1-MMP (60 kDa)
β-actin
MMP-2 (63+72 kDa)
β-actin
N-cadherin (140 kDa)
β-actin

c

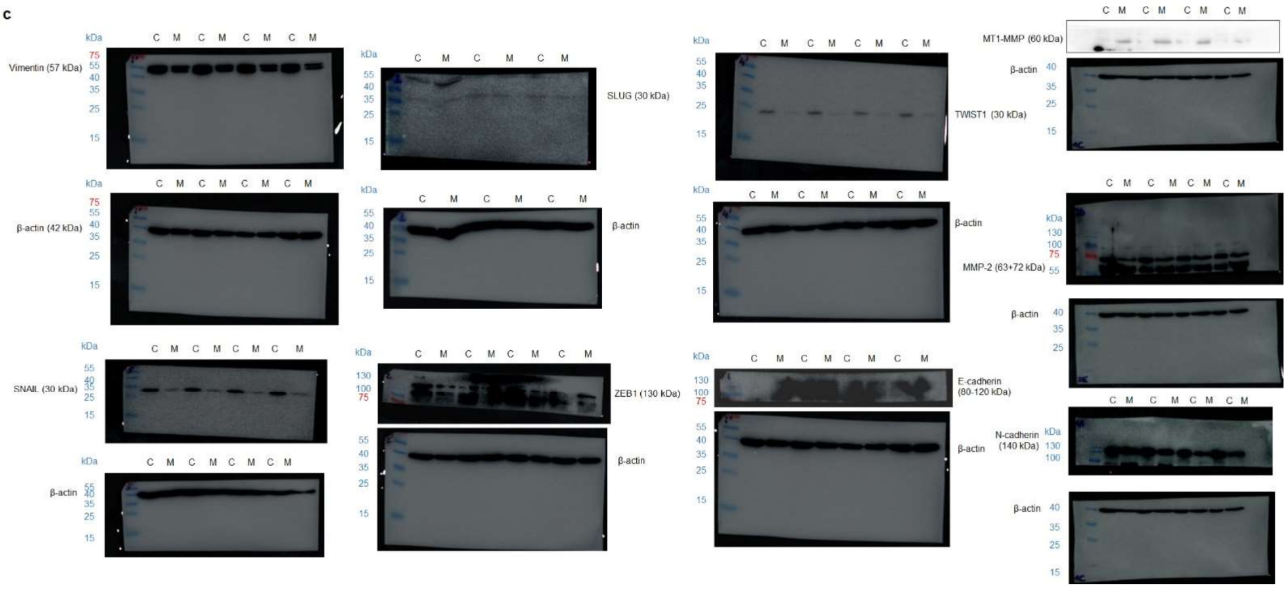

Figure S4. Uncropped western blot images. Shown are whole western blot (WB) membranes used for the determination of the effects of MitoQ on the expression of EMT markers by human breast cancer cells. Note that, in order to be able to show molecular ladders, the membranes are shown at the longest exposure time (often overexposed), which does not always correspond to the acquisition time on an ECL imager 600. (**a**) WB of MDA-MB-231 cancer cells corresponding to Figure 4d. (**b**) WB of SkBr3 cancer cells corresponding to Figure 4f. (**c**) WB of MDA-MB-436 cancer cells corresponding to Figure 4h. Note that some blots were stripped and reprobed. In that case, the same β-actin bands were used for controls. Abbreviation: C, control; M, MitoQ.

Table S1. Antibodies used for western blotting and immunofluorescence.

Protein Name	Antibody	Company & Catalogue #	Dilution
Vimentin	rabbit anti-vimentin	Cell signaling, #5741	1:1,000
Vimentin*	mouse anti-vimentin	Dako, #M072529-2	1:100
SNAIL	rabbit anti-snail	Cell signaling, #3879	1:1,000
SLUG	rabbit anti-slug	Cell signaling, #9585	1:1,000
ZEB1	rabbit anti-zeb1	Cell signaling, #3396	1:1,000
TWIST1	rabbit anti-twist1	Cell signaling, #46702	1:1,000
E-cadherin	rabbit anti-E-cadherin	Cell signaling, #3194	1:1,000
E-cadherin*	mouse anti-E-cadherin	Cell signaling #14472	1:50
N-cadherin	rabbit anti-N-cadherin	Cell signaling #13116	1:1000
MT1-MMP	mouse anti-MT1-MMP	Chemicon #MAB1767	1:1000
MMP-2	rabbit anti-MMP-2	Santa Cruz, #sc-10736	1:1000
β-actin	mouse anti-β-actin	Sigma-Aldrich, #A5441	1:10,000

* antibodies used in immunofluorescence.

Table S2. Primers used in quantitative PCR.

Human gene name	Forward 5′ - 3′	Reverse 5′- 3′
VIM	CGGGAGAAATTGCAGGAGGA	AAGGTCAAGACGTGCCAGAG
SNAI1	AATCCAGAGTTTACCTTCCAGCA	TCCCAGATGAGCATTGGCAG
SNAI2	GAACTGGACACACATACAGTGAT	ACAGTGATGGGGCTGTATGC
ZEB1	GCCCAAACTGCAAGAAACGC	GTCGCCCATTCACAGGTATCA
TWIST1	TTCTCGGTCTGGAGGATGGA	TCTCTGGAAACAATGACATCTAGG
c-MYC	ATGAAAAGGCCCCCAAGGTA	TTTCCGCAACAAGTCCTCTTC
OCT4	CATCAAAGCTCTGCAGAAAGAAC T	CTGAATACCTTCCCAAATAGAACC C
NANOG	AATACCTCAGCCTCCAGCAGATG	TGCGTCACACCATTGCTATTCTTC
SOX2	CGAGTGGAAACTTTTGTCGGA	TGTGCAGCGGCTCGCAG
β-ACTIN	CCCGCGAGCACAGAGC	TCATCATCCATGGTGAGCTGG
